# Announcing the Availability of a Culture Collection of Uranium-Resistant Microbial Assemblages (CURMA) Obtained from Metalliferous Soils of the Savannah River Site, USA

**DOI:** 10.1128/MRA.00551-20

**Published:** 2020-07-23

**Authors:** Meenakshi Agarwal, Rajesh Singh Rathore, Allen Black, Xiaoyu Xu, John Seaman, Ashvini Chauhan

**Affiliations:** a School of the Environment, Florida A&M University, Tallahassee, Florida, USA; b Savannah River Ecology Laboratory, University of Georgia, Aiken, South Carolina, USA; Indiana University, Bloomington

## Abstract

Metagenomic assessment provides a comprehensive survey of soil microbiota; however, isolation and characterization of functionally relevant microbiota are required prior to their application(s), such as for metal remediation. Toward this end, we report the availability of a culture collection comprising uranium (U)-resistant microbial assemblages (CURMA) to the scientific community.

## ANNOUNCEMENT

Uranium (U) is a predominant radionuclide contaminant present in long-term-contaminated soils, such as at the Savannah River Site (SRS), a former nuclear legacy site located along the Savannah River near Aiken, South Carolina (1). Microbial communities that exist in heavy metal-rich soils have been shown to develop various mechanisms to resist and bioremediate U (2, 3). The application of culture-independent approaches, such as metagenomics, has significantly enhanced our knowledge of the diversity of microbial communities colonizing different ecosystems (4). However, the isolation of U-resistant microbes using culture-dependent approaches, including culturomics (5), is a prerequisite to better understanding the microbially mediated bioremediation processes and *in situ* application of obtained microbiota. Toward this end, this article reports on the availability of a culture collection consisting of uranium (U)-resistant microbial assemblages (CURMA) (pronounced “karma”), which represents a plethora of U-resistant bacterial and fungal strains resistant to variable levels of uranium.

Soil samples for this study were collected from metalliferous SRS 101 (33°19′02.1ʺN, 81°42′54.0ʺW) and shipped overnight on ice to the Florida A&M University (FAMU) laboratory. Soil was homogenized, serially diluted, and plated on lysogeny broth (LB) agar, *Bradyrhizobium* selective medium (BJSM), and potato dextrose agar (PDA) supplemented with U (2 mM) in the form of uranyl nitrate, followed by incubation at 30°C, as reported previously (6). Colonies with variable morphologies were selected and streaked onto 2 mM U to obtain isolated colonies. An individual colony of each isolate was inoculated in liquid medium and incubated at 30°C until growth occurred. Isolated strains were frozen in a solution of autoclaved 15% glycerol and preserved at −80°C. DNA was extracted from the isolates using the ZR fungal/bacterial DNA kit (Zymo Research, Irvine, CA, USA) and identified using 16S rRNA and 18S rRNA gene sequencing, as shown before (6). The obtained 16S and 18S rRNA gene sequences were analyzed using NCBI BLAST, and details are presented in Table 1. These isolates were further analyzed to determine the MIC against U using our recently developed plate MIC method (6).

**TABLE 1:** Bacterial and fungal strains represented in CURMA, identified by 16S and 18S rRNA gene sequencing

Strain identifier	Species^*a*^	U MIC value (mM)	NCBI accession no.
SRS-1-W-2018	*Chromobacterium vaccinii	7	MT254579
SRS-2-W-2018	*Serratia marcescens	7	MT254580
SRS-11-W-2018	*Pseudomonas chengduensis	6	MT322934
SRS-8-S-2018	*Serratia marcescens	6	MT322935
SRS-9-S-2018	*Serratia marcescens	6	MT322936
SRS-18-S-2018	*Bacillus sp.	6	MT322937
SRS-19-S-2018	*Lysinibacillus sp.	6	MT322938
SRS-20-S-2018	*Pseudomonas umsongensis	7	MT322939
SRS-21-S-2018	*Bacillus sp.	6	MT322940
SRS-22-S-2018	*Bacillus megaterium	6	MT322941
SRS-41-S-2018	*Burkholderia contaminans	7	MT322942
SRS-54-S-2018	*Pseudomonas vancouverensis	6	MT322943
SRS-88-S-2018	*Pseudomonas sp.	5	MT322944
SRS-104-S-2018	*Bacillus sp.	5	MT322945
SRS-115-S-2018	*Paenibacillus dendritiformis	2	MT322946
SRS-146-S-2018	*Burkholderia glumae	8	MT322947
SRS-147-S-2018	*Burkholderia glumae	8	MT322948
SRS-120-S-2019	Acinetobacter guillouiae	2	MT322949
SRS-122-S-2019	Bacillus megaterium	2	MT322950
SRS-123-S-2019	Bacillus firmus	2	MT322951
SRS-124-S-2019	*Bacillus* sp.	2	MT322952
SRS-125-S-2019	Bacillus cereus	2	MT322953
SRS-126-S-2019	Acinetobacter guillouiae	2	MT322954
SRS-127-S-2019	Pseudomonas helmanticensis	2	MT322955
SRS-128-S-2019	*Sporosarcina* sp.	2	MT322956
SRS-151-F-2019	*Bacillus* sp.	2	MT322957
SRS-157-F-2019	*Bacillus* sp.	2	MT322958
SRS-158-F-2019	*Kinneretia* sp.	2	MT322959
SRS-159-F-2019	*Aeromonas* sp.	2	MT322960
SRS-160-F-2019	Kosakonia radicincitans	2	MT322961
SRS-162-F-2019	*Kosakonia* sp.	2	MT322962
SRS-163-F-2019	Bacillus marisflavi	2	MT322963
SRS-178-F-2019	Curvibacter gracilis	2	MT322964
SRS-179-F-2019	*Curvibacter* sp.	2	MT322965
SRS-181-F-2019	*Ralstonia* sp.	2	MT322966
SRS-182-F-2019	Ralstonia pickettii	2	MT322967
SRS-183-F-2019	Ralstonia pickettii	2	MT322968
SRS-184-F-2019	Roseateles terrae	2	MT322969
SRS-185-F-2019	Roseateles terrae	2	MT322970
SRS-187-F-2019	*Bradyrhizobium oligotrophicum	2 mM	MT322971
SRS-189-F-2019	*Bradyrhizobium oligotrophicum	2 mM	MT322972
SRS-190-F-2019	*Bradyrhizobium sp.	2	MT322973
SRS-191-F-2019	*Bradyrhizobium sp.	2	MT322974
SRS-6-S-2018	*Penicillium limosum	25	MT328140
SRS-7-S-2018	*Penicillium limosum	20	MT328141
SRS-32-S-2018	Talaromyces leycettanus	2	MT328142
SRS-33-S-2018	Paraphaeosphaeria viciae	2	MT328143
SRS-34-S-2018	*Antrodia* sp.	2	MT328144
SRS-35-S-2018	Purpureocillium lilacinum	2	MT328145
SRS-36-S-2018	Purpureocillium lilacinum	2	MT328146
SRS-37-S-2018	Pyrenochaetopsis leptospora	2	MT328147
SRS-38-S-2018	Pyrenochaeta nobilis	2	MT328148
SRS-39-S-2018	Diaporthe maritima	2	MT328149
SRS-40-S-2018	*Penicillium limosum	20	MT328150
SRS-45-S-2018	*Rhodotorula sp.	6	MT328151
SRS-62-S-2018	*Penicillium limosum	18	MT328152
SRS-63-S-2018	*Penicillium limosum	18	MT328153
SRS-64-S-2018	*Penicillium limosum	20	MT328154
SRS-65-S-2018	Talaromyces leycettanus	2	MT328155
SRS-66-S-2018	*Megasporia* sp.	2	MT328156
SRS-67-S-2018	*Penicillium limosum*	2	MT328157
SRS-68-S-2018	*Penicillium limosum*	2	MT328158
SRS-69-S-2018	*Albifimbria* sp.	2	MT328159
SRS-71-S-2018	Trichoderma lixii	2	MT328160
SRS-96-S-2018	Sugiyamaella smithiae	2	MT328162
SRS-97-S-2018	Candida labiduridarum	2	MT328163
SRS-21-S-2019	*Aspergillus versicolor	8	MT328172
SRS-168-F-2019	Lepidosphaeria nicotiae	2	MT328166
SRS-169-F-2019	*Arthrinium* sp.	2	MT328167
SRS-170-F-2019	*Lepidosphaeria nicotiae*	2	MT328168
SRS-171-F-2019	Didymosphaeria variabile	2	MT328169
SRS-172-F-2019	*Lepidosphaeria nicotiae*	2	MT328170

a Note that all strains were isolated on plates containing 2 mM uranium and hence are U resistant. Some strains, designated by an asterisk (*), were chosen for additional MIC assessment based on their predominance in the metalliferous SRS soils (5, 9, 10).

Overall, we isolated a diverse group of U-resistant bacterial and fungal strains (Table 1), with *Bacillus* spp. (*n* = 11) and *Penicillium* spp. (*n* = 8) being dominant; some of these groups have also been identified as predominant groups in metagenomic analyses (7, 8), including our work on this aspect (5, 9, 10). Some other groups retrieved as higher representatives included *Pseudomonas* (*n* = 4), *Bradyrhizobium* (*n* = 4), *Burkholderia* (*n* = 3), *Serratia* (*n* = 3), and *Lepidosphaeria* (*n* = 3). Notably, among the tested microbial strains, fungal isolates exhibited a higher resistance to U than did the bacterial strains (Table 1). In summation, this article reports on the availability of CURMA (collection of uranium-resistant microbial associates), which is represented by both bacterial and fungal organisms that are resistant to U and are available to the scientific community for their research needs. The availability of this collection will continue to enhance our understanding of microbial mechanisms for U resistance and bioremediation.

### Data availability.

The 16S and 18S rRNA gene sequences obtained from this research were deposited in NCBI, and the accession numbers are listed in Table 1. CURMA isolates are available upon request and can be shipped as an axenic bacterial/fungal strain(s) inoculated onto agar plates or as slants using the growth conditions described herein.
